# Utilization of Waste Polysilicon Sludge in Concrete

**DOI:** 10.3390/ma13010251

**Published:** 2020-01-06

**Authors:** Abdul Qudoos, In Kyu Jeon, Seong Soo Kim, Jeong Bae Lee, Hong Gi Kim

**Affiliations:** 1 Civil Engineering Department, Balochistan University of Engineering and Technology, Khuzdar 89100, Pakistan; qudoos.engnr@gmail.com; 2 Department of Civil and Environmental Engineering, Hanyang University, 222, Wangsimni-ro, Seongdong-gu, Seoul 04763, Korea; supermacy94@daum.net; 3 Department of Civil Engineering, Daejin University1007, Hoguk-ro, Pocheon-si 11159, Korea; sskim@daejin.ac.kr; 4 GFC R&D CO., Ltd., 155 Jajak-ro, Pocheon-si, Gyenoggi-do 11158, Korea; dlwjdqo@nate.com

**Keywords:** concrete, waste, polysilicon sludge, compressive strength

## Abstract

Increasing use of cement in the construction industry is causing an alarming increase in carbon dioxide (CO_2_) emissions, which is a serious environmental threat, it can be reduced by the addition of supplementary cementitious materials (SCMs). The commonly used SCMs like ground granulated blast furnace slag (GGBS), metakaolin (MK) and fly ash (FA) have been successfully used to replace the cement partially or completely. Polysilicon sludge obtained from the photovoltaic industry is also a type of waste material that can be used as SCM because it has high content of reactive SiO_2_. This study investigates the effects of replacing cement with polysilicon sludge in concrete. Different concrete specimens were made by replacing varying proportions of cement with polysilicon sludge and their properties, such as, fresh properties, compressive strength, heat release, chloride penetration, freeze/thaw resistance and microstructural investigations were determined. The results demonstrate that the polysilicon sludge can be used effectively to replace cement, and environmental threats associated with its disposal can be reduced.

## 1. Introduction

Rapid increase in use of concrete throughout the world has become a serious menace to the green environment. Cement is one of main constituents of concrete, and its manufacture is linked with the emission of greenhouse gases, specifically CO_2_. Approximately, 0.8 kg CO_2_ is emanated in the atmosphere during the production of 1.0 kg of cement clinker [1,2,3], with cement production accounting for 6–7% of worldwide CO_2_ emissions [1,3]. Hence, many efforts have been made to diminish excessive utilization of cement in the concrete industry to minimize the greenhouse gas emissions [4]. For this purpose, researchers have suggested to increase the incorporation of SCMs as possible replacement of cement. The frequently recycled SCMs, like, silica fume [5], FA [6], and MK [7] have been effectively added to substitute cement partially or completely. These materials exhibit pozzolanic activity; the compounds making up these materials react with hydration products of cement, resulting in the development of surplus calcium-silicate-hydrate (C-S-H) gel. This behavior improves the overall performance of cementitious composites. Previously, various types of discarded/waste materials have been considered as partial replacement of cement in concrete [8,9,10]. In terms of environmental and economic aspects, they are attractive solutions for energy savings and waste minimization in the construction industry [11]. The use of silica-rich materials is another potential solution to partially replace cement [12,13]. Recent studies also reveal that waste glass powder, rich in silica, can be used as cement replacement too [14]. Silica fines, such as micro-silica, fumed silica, silica flour, and nano-silica are primarily composed of high content of pure SiO_2_ with extremely small particle sizes [12]. 

In the photovoltaic (PV) industry, the solar cell technology is well-known, and is a viable and reliable source for clean electrical energy [15]. It accounts for over 1 billion (US Dollars) in yearly worldwide sales. The PV industry is anticipated to increase production capacity by tens of gigawatts (GW) per year [16]. Solar-grade silicon i.e., polysilicon (PS) is most comprehensively used material in the photovoltaic industry, which is experiencing large growth [17,18,19,20]. The PS sludge produced during refining of PV solar panels is another possible source of nano-silica powder [21]. In the Republic of Korea, 50,000 tons of PS sludge is being produced annually [12]. Inappropriate discarding of the enormous quantities of PS sludge generated annually in South Korea is catastrophic. The dumping places are near to reservoirs or groundwater sources. Thus, PS nano-particles could contaminate drinking water and resulting in heart and lung-related diseases. Humans are susceptible to diseases related to the nano-particles existing in PS sludge; hence, methods for disposal of hazardous sludge by immobilization and consolidation within a useful resource are highly required. In addition, interest in sustainable development and awareness of eco-friendly concrete is increasing with global population growth. The use of PS sludge thus can result in environmentally-friendly and sustainable construction material. 

PS is the basic component of crystalline silicon modules. Various techniques are used to solidify and melt PS for production of ingots with different degrees of crystallinity [22]. The most important crystallization technology is the directional solidification process. Single silicon crystals are developed by the Czochralski (Cz) process, and polycrystalline silicon is produced by casting [22,23]. Ingots manufactured by both techniques are sliced into thin wafers using a wire saw and processed into solar cells. During wafer processing, a silicon layer approximately 250 µm thick is wasted per wafer [22]. 

The feasibility for using waste sludge from the semiconductor industry in cement systems has been investigated by various researchers [24,25,26,27,28]. However, utilization of waste sludge from PV panels has not been thoroughly investigated. It is imperative to explore PV waste sludge to address environmental concerns related to human health. For this purpose, this investigation was conducted to study the possibility of using PS sludge as a SCM. Concrete specimens were fabricated by substituting varying proportions of cement with polysilicon sludge, and investigated for fresh properties, compressive strength, heat flow, chloride penetration and freeze/thaw resistance and microstructural characteristics. The microstructure of pastes and concretes was investigated by X-ray diffraction, infrared spectroscopy and scanning electron microscopy. 

## 2. Materials and Methods 

### 2.1. Materials

Ordinary Portland cement (OPC) in compliance to ASTM C150, and polysilicon sludge cake from wet waste acquired from O Ceramic Company (Gunsan, Korea) were used as binders to make concrete specimens. After drying the wet samples at 105 °C for 24 h, refined PS was obtained by pulverization. The chemical composition of PS was determined by X-ray fluorescence (XRF) technique. The detailed chemical composition, expressed in terms of oxides and physical characteristics of OPC and PS are given in Table 1. Aggregates were obtained from Gunsan, Korea, and their physical properties are presented in Table 2. In addition, a poly-carboxylic superplasticizer (SP) and air entraining admixture (AE) (properties presented in Table 3) were used. Air entraining admixture was added in order to improve the resistance of concrete mixes in a freezing/thawing environment [29]. 

### 2.2. Mix Proportions and Methodology

The particle size distribution (PSD) of PS was investigated with a particle size analyzer (Malvern Mastersizer 2000, Malvern, Malvern, UK). The mineralogical composition of PS was determined by X-ray diffractometer (model: RINT D/max 2500, 40 Kv, 30 Ma, Rigaku, Tokyo, Japan). To detemine the morphology of PS, a scanning electron microscope (SEM) analysis was done with a SEM analyzer (model: Philips XL30ESEM, FEG, Hillsboro, OR, USA). 

The concrete mixes were designed with a mean target strength of 35.0 MPa and 120 mm slump. Six different mix proportions were produced by replacing 0.0, 5.0, 10.0, 15.0, 20.0, and 25.0% of OPC with PS by weight, whereas, the water/binder (W/B) ratio sand/total aggregate (S/A) ratio were fixed at 0.40 and 0.48 as shown in Table 4, respectively. Fresh properties i.e., air content and slump of the concrete mixes were examined. Paste specimens were made using a W/B ratio of 0.40 and investigated for heat flow using a semi-adiabatic calorimeter [30,31]. For Fourier transform infrared spectroscopy(FTIR) and XRD analyses, paste specimens were made using a W/B ratio of 0.4 and cured for 3, 7, and 28 days. After each curing age, powder specimens were obtained, treated with acetone and dried before analysis. The cylindrical concrete specimens of sizes (ϕ 100 × 200 mm^2^) were prepared, covered with plastic sheets and kept in room temperature for 24 h, and after 24 h, they were demolded and were submerged in water at 20 ± 2 °C for curing for 3, 7 and 28 days curing.

The compressive strength of three replicate specimens from each mix were determined after curing of 3, 7 and 28 days according to ASTM C 109, with a universal testing machine (model: CCM-200A; Shimadzu Corporation, Kyoto, Japan). Moreover, after 28 days, chloride penetration and accelerated freeze/thaw tests were conducted according to NT BUILD 492 [32] and ASTM C 666 [33], respectively. Dynamic modulus of elasticity of concrete specimens exposed to accelerated freeze/thaw test were evaluated following ASTM C 215. Relative dynamic modulus of elasticity was calculated using the equation:

P_c_ = (n_c_^2^/n^2^) × 100 (1)

where: P_c_ = relative dynamic modulus of elasticity, after c cycles of freezing and thawing, percent; n = fundamental transverse frequency at 0 cycles of freezing and thawing, and n_c_ = fundamental transverse frequency at c cycles of freezing and thawing.

For FTIR, (Nicolet Is50, Thermo Fisher Scientific, Waltham, MA, USA) and X-ray diffraction (XRD) analyses, acetone-treated and desiccated/dried powder samples were obtained after 3, 7, and 28 days of hydration from the paste specimens. In addition, thin samples for SEM analysis were acquired and engrossed in alcohol for 24 h before drying them in an oven at 60 °C, as previously described by [34].

## 3. Results and Discussion

### 3.1. Properties of Polysilicon Sludge

**Figure 1:**
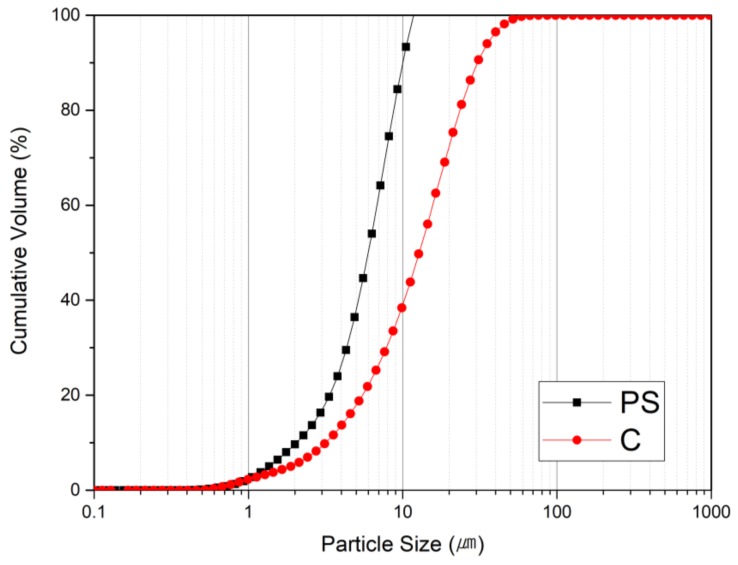
Particle size distribution curves of polysilicon (PS) and ordinary portland cement (OPC) samples.

**Figure 2:**
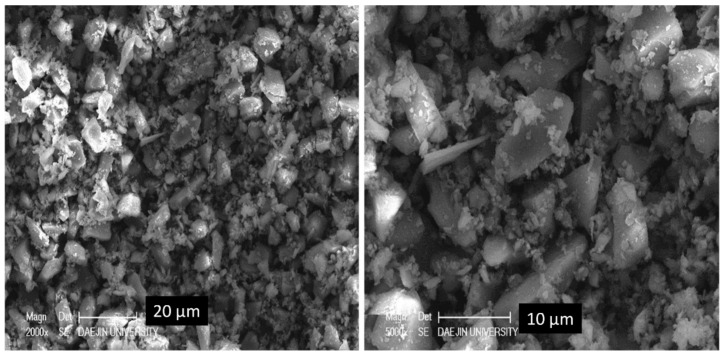
Morphology of PS particles.

**Figure 3:**
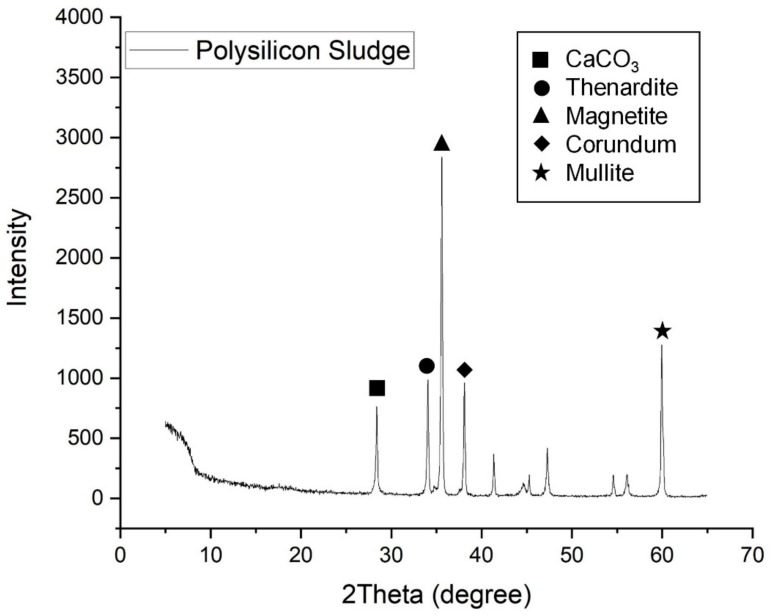
XRD patterns of PS particles.

The particle size distribution (PSD) of the PS sludge and OPC are shown in Figure 1. As can be seen the PS sludge have finer particles than the OPC specimen. Table 1 summarizes the chemical compositions of the OPC and PS specimens. The major components of PS were SiO_2_ (96%) and Fe_2_O_3_. The high silica content and high specific area suggest pozzolanic properties. Figure 2 shows the morphology of PS particles obtained by SEM. Particle size distribution of PS can be witnessed with micro- and nano-size range particles, which are agglomerated with angular, irregular, and of spherical shapes. Figure 3 exhibits the XRD patterns of the PS particles, showing that the silica is present in the PS specimens in amorphous form.

### 3.2. Fresh Concrete Properties

**Figure 4:**
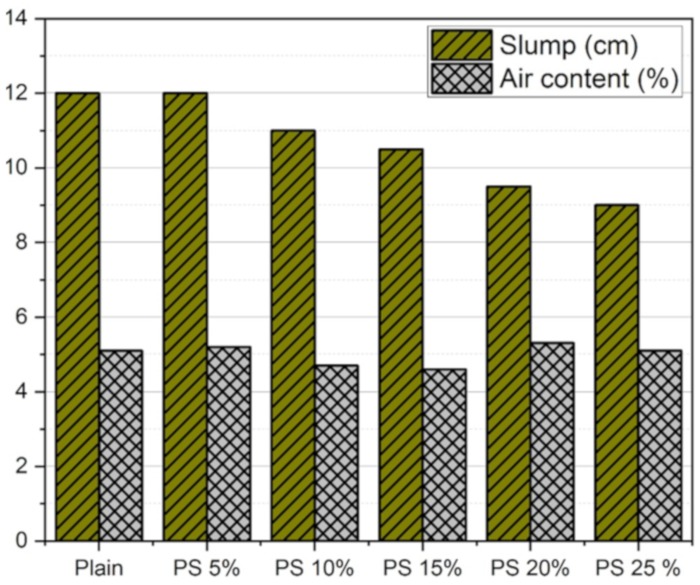
Results of slump and air content tests.

Figure 4 displays the results of slump and air content with each mix design. The addition of PS particles caused a reduction in the slump values. It may be due to the reason that the specific surface area of PS particles is higher than that of the cement particles. On the other hand, the air content of all the specimens were similar, indicating that PS does not have a greater impact on air entrainment of the concrete matrix. 

### 3.3. Compressive Strength

**Figure 5:**
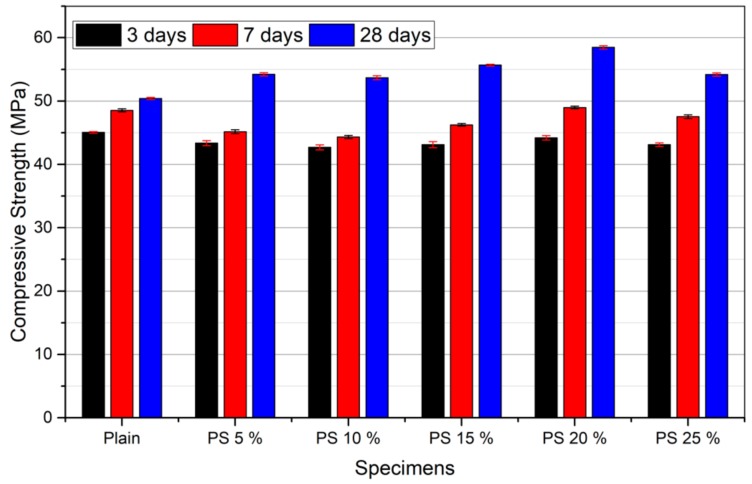
Compressive strength test results.

The results of compressive strength test after 3, 7, and 28 days are presented in Figure 5. Pozzolanic activity index (PAI) was calculated following ASTM C 311 and summarized in Table 5. At 3 days, the strengths of the plain sample and PS samples are nearly the same. Compressive strength of the plain specimens at 7 and 28 days increased by 4 and 17%, respectively, compared to that of the specimens at 3 days. On the other hand, the PS sample with 5% replacement showed an increased strength of 12 and 20% after 7 and 28 days, respectively, in comparison with that at 3 days. Similar trend was observed for other specimens. Generally, an increase of 2–9% and 6–14% in compressive strength was observed for the specimens containing PS particles compared to that of the plain specimen at 7 and 28 days, respectively. The relatively lower compressive strength of PS samples at early stage may be attributed to the low pozzolanic activity of PS particles at the early age. Table 5 indicates that all the concrete mixes demonstrated a PAI value greater than 75%, which is the least limit for pozzolanicity of a material according to ASTM C 618. The chemical reaction of OPC can be described as follows [35]:

2C_3_S + 6H_2_O (H) → C-S-H + 3Ca(OH)_2_ (CH), 2C_2_S + 4H_2_O (H) → C-S-H + Ca(OH)_2_ (CH), (2)

Whereas, the pozzolanic reaction is defined as below [5,36,37]:

Ca(OH)_2_ (CH) + SiO_2_ + H_2_O (H) → C-S-H gel. (3)

As presented by the above reactions, calcium hydroxide (CH) is produced by the hydration reaction of OPC and contributes to pozzolanic reactions. The pozzolanic reaction is secondary reaction which occurs only after onset of hydration of the OPC [35]. However, PS reacts with CH in secondary reaction. This pozzolanic reaction can improve the microstructure by increasing density with consumption of CH and development of C-S-H phases [38,39]. Thus, these characteristics of PS could have led to the improvement in compressive strength with time. It is important to note here that the specimens which presented considerable variations in the compressive strength test results (Plain, PS 10%, and PS 20%) were selected for further analyses.

### 3.4. Heat Release Profiles

**Figure 6:**
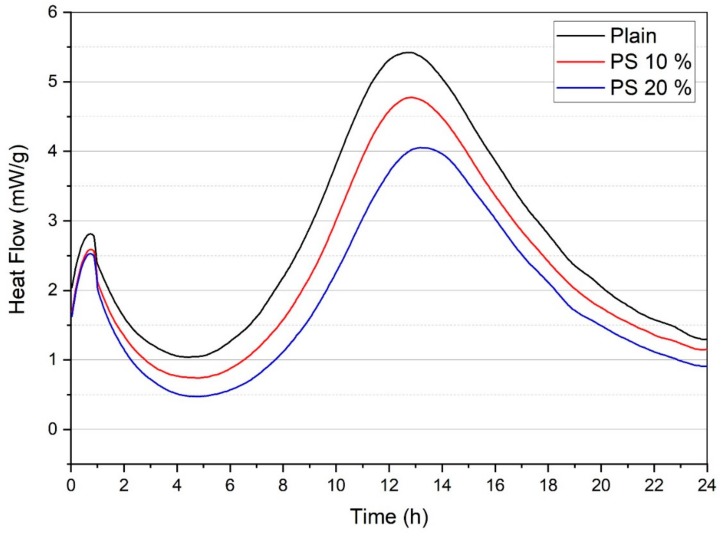
Heat flow of PS and OPC paste.

**Figure 7:**
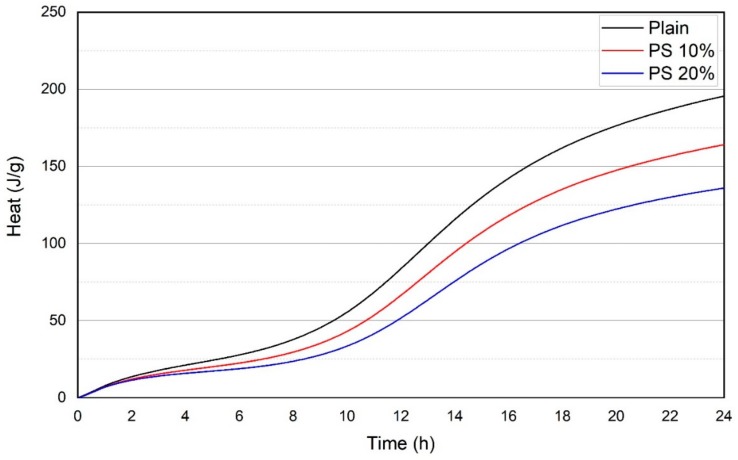
Total heat evolution of PS and OPC pastes.

The heat flow measurements of samples containing PS are shown in Figure 6. The typical stages of early hydration of cementitious systems consist of preliminary dissolution of cement, dormant period, main hydration due to C_3_S reaction, sulfate depletion, and accelerated aluminate activity [40]. The initial peak, with considerably high heat flow, corresponds to early dampening and termination of raw materials, whereas the subsequent peak is accredited to quickening of the reaction development and formation of substantial C-S-H gel [41]. It can be seen in Figure 6 that the plain specimens demonstrated highest peak for heat flow curves compared to the specimens containing PS particles. This can be accredited to the dilution effect of cement. Additionally, the low heat evolution for the PS containing specimens may be attributed to the slow pozzolanic reaction of PS particles at the early age. Figure 7 displays the total heat profiles for various specimens. The total heat released by PS 10% and PS 20% decreased by 3.6% and 9.7%, respectively, compared to that of the plain sample. 

### 3.5. Chloride Penetration

**Figure 8:**
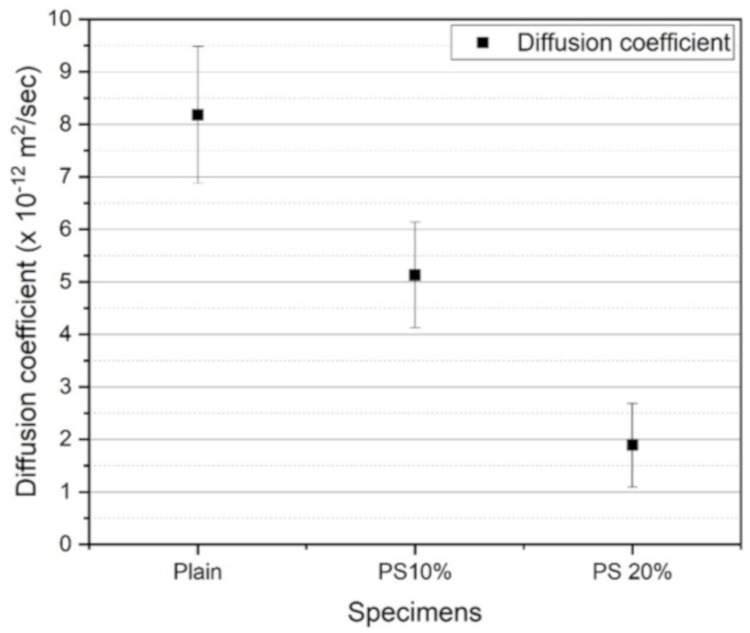
Chloride diffusion coefficients for various specimens at 28 days.

Figure 8 depicts the results of chloride penetration test in terms of diffusion coefficients. The results showed a similar tendency to that of the compressive strength test. The incorporation of PS particles enhanced the chloride penetration resistance of the specimens. The increased chloride penetration resistance of the specimens containing PS is attributed to microstructure enhancement due to the secondary pozzolanic reaction [42]. In addition, the interfacial transition zone (ITZ) could have become denser than that of the plain sample due to addition of PS and the subsequent pozzolanic reactions. There are three well-known diffusion paths of chloride ions into concrete: interlocked pores in cement pastes, interconnected pores in aggregates, and connected pores in the ITZ [43,44]. The permeability within the aggregate is considerably inferior to those of the hydrated paste and ITZ. Therefore, the main diffusion paths exist in the concrete paste and ITZ. PS with fine particles and pozzolanic properties could induce greater production of C-S-H phases near the ITZ [45]. 

### 3.6. Freeze/Thaw Resistance

**Figure 9:**
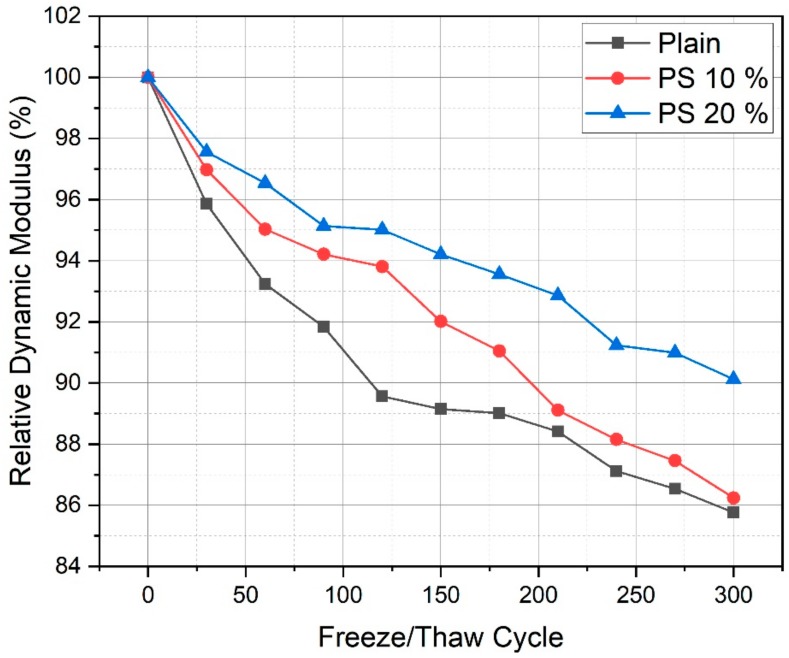
Results of relative dynamic modulus of elasticity after 300 freezing and thawing cycles.

**Figure 10:**
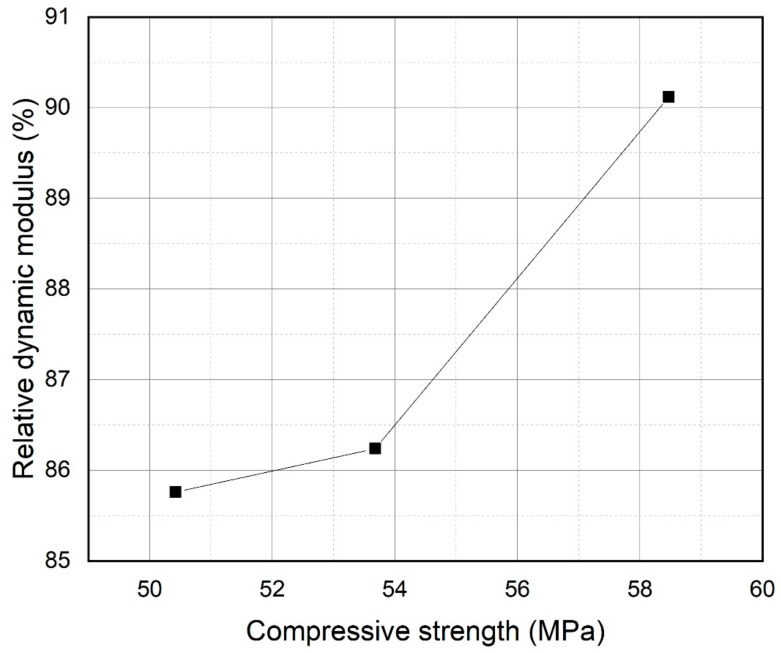
Relationship of compressive strength and relative dynamic modulus.

Figure 9 presents the relative dynamic modulus of elasticity of samples after 300 freezing and thawing cycles. A relative dynamic modulus value greater than 60% was considered satisfactory. However, values under 60% indicated very low frost resistance. The plain sample had a relative dynamic modulus of elasticity of 85.76%, however, the PS samples showed improved frost resistance relative to the plain sample. In particular, the PS 20% sample had a relative dynamic modulus value of 90.12% after 300 cycles. The relative dynamic modulus of the plain sample decreased by 44% more than that of the PS 20% sample. When concrete is exposed to frost conditions, ice development in the capillary pores of the concrete usually generates expansion stress. The presence of entrained air can reduce the expansion stress that is caused by the creation of ice capillary pores [46,47,48,49]. Therefore, to protect damage by freezing and thawing, it is important to control the entrained air. This could cause reduced capillary pore volumes. For this purpose, it can be presumed that the capillary pores in PS samples are reduced or that the entrained air in PS samples increased the frost resistance of PS samples. It was shown that PS samples had sophisticated resistance to freezing-thawing than plain sample. As aforementioned in the compressive strength section, the PS samples showed higher compressive strength at later stages due to pozzolanic reactions and the fine particle size of PS. Figure 10 displays the comparison of compressive strength at 28 days and relative dynamic modlus of elasticity after 300 cycles for plain, PS 10%, and PS 20% concrete specimens. It can be seen that increasing that the specimens which presented higher compressive strength resulted in higher relative elastic modulus. Thus, it could be inferred that the capillary pores in samples were filled by C-S-H phases. In addition, the fine particles of PS could result in a relatively dense cement matrix compared to the plain sample. Therefore, introduction of PS into concrete cement systems could improve frost resistance. 

### 3.7. X-Ray Diffraction Analysis

**Figure 11:**
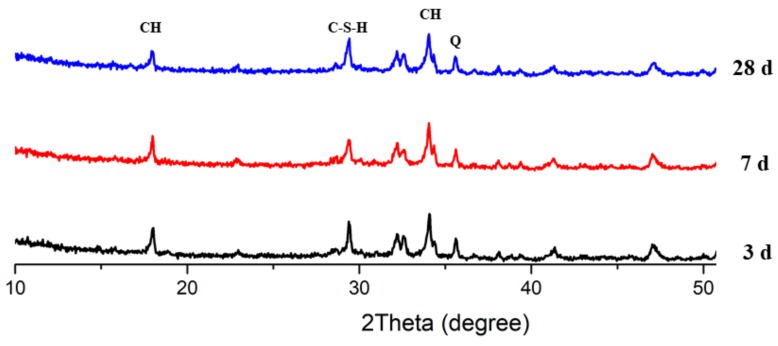
XRD patterns of plain samples.

**Figure 12:**
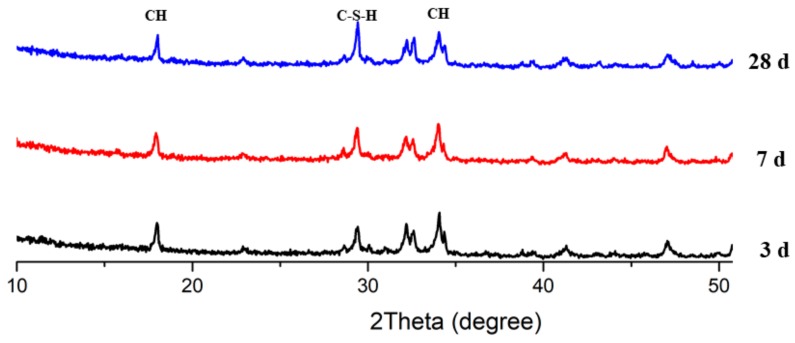
XRD patterns of mix PS 10%.

**Figure 13:**
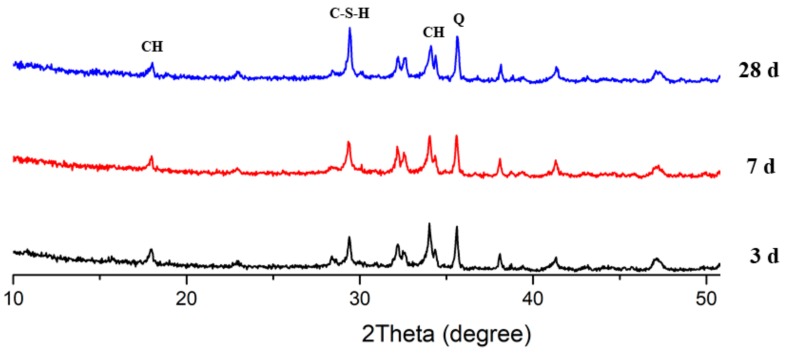
XRD patterns of mix PS 20%.

Figure 11, Figure 12 and Figure 13 present the XRD patterns of three different mixes, which include the plain, PS 10%, and PS 20% samples after 3, 7, and 28 days, respectively. Various peaks emerged at various ages. The intensity peaks of CH can be found at 18.02° and 34.04° in the XRD patterns, which is one of the leading hydration products. The intensity of the peaks for CH decreased with increasing curing time. With all the samples, the highest peak intensity of CH was present at 3 days. Additionally, inclusion of PS caused a reduction in CH peak height while increasing the peak for C-S-H, indicating the consumption of CH in the secondary reaction with PS to produce C-S-H phases. 

### 3.8. Fourier-Transform Infrared Spectroscopy

**Figure 14:**
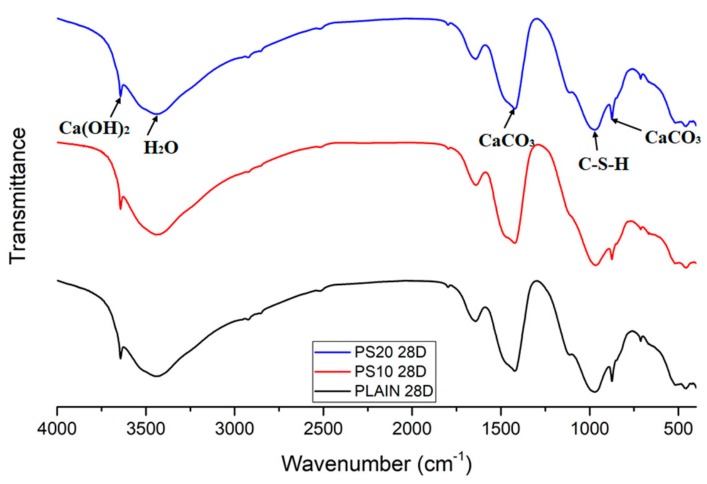
FTIR spectra of specimens after 28 days.

The IR spectra for various mix specimens at 28 days are presented in Figure 14. All the spectra seem identical, however, the intensity and position of the peaks are different. The FTIR bands at 3642, 3400–3100, 640 cm^−1^are indorsed to O–H stretching of CH, v1 and v2 stretching of the water molecules, v2 bending of the water molecules, respectively. Whereas, the bands at 970 and 870 cm^−1^ are associated with Si–O stretching of polymeric C-S-H, and v2-stretching of carbonate, respectively [42,50,51]. 

Apart from this, the doublet bands at 1680 and 1432 cm^−1^ are liked with the C–O stretching of the CO_3_^2−^ [51]. Whereas, the absorption bands can be witnessed in the range 1100–1200 cm^−1^ that are because of the v3 stretching vibrations of SO_4_^2−^, signifying the existence of ettringite and gypsum [52]. The magnitude of the sharp absorption peak at 3642 cm^−1^ diminished as the cement was partially substituted with PS particles. This demonstrates the degree of ingesting of portlandite due to dilution or pozzolanic effects. The IR bands for a number of specimens emerged at the equivalent locations with varying intensities, which are primarily associated to development of hydration products, like, CH and C-S-H [42]. The wide-ranging bands due to water molecules are positioned at 3450 cm^−1^ that became smaller and wider with inclusion of PS particles. This phenomenon indicates decreases in bonded –OH groups and free water [53]. The IR bands around 876, 1432 and 1480 cm^−1^ specify carbonation in the paste samples [42].

### 3.9. Scanning Electron Microscopy

**Figure 15:**
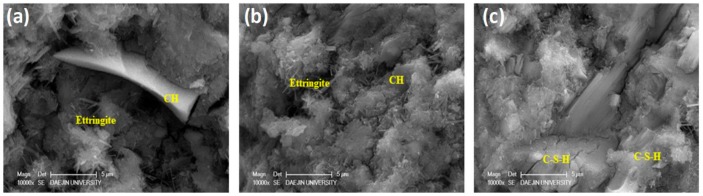
SEM micrographs of plain sample at (**a**) 3 days, (**b**) 7 days, and (**c**) 28 days.

**Figure 16:**
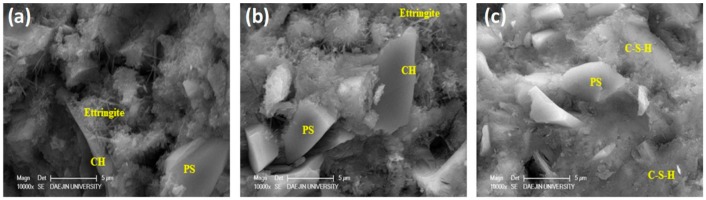
SEM micrographs of PS 20% sample at (**a**) 3 days, (**b**) 7 days, and (**c**) 28 days.

Microstructures of the plain specimen and the specimens containing 20% PS were investigated using SEM at various ages, as shown in Figure 15 and Figure 16. The plain specimens demonstrated a relatively porous microstructure compared to the specimens containing 20% PS at their respective ages. CH crystals were large and plate-like, while the dark areas represent voids. In contrast, incorporation of PS particles densified the microstructure owing to the pozzolanic effect [54]. These results clearly prove that integration of PS particles improved the microstructure of concrete specimens.

## 4. Conclusions

This study explored the effects of replacing cement with polysilicon sludge particles on physical, mechanical, and chemical properties of concrete. Based on the obtained consequences, following conclusions are made.

1. Polysilicon sludge particles are finer than cement particles and contain a large amount of SiO_2_ in amorphous form.
2. The incorporation of PS particles as a cement replacement material reduced the early heat of hydration, suggesting a slower reaction rate of PS particles.
3. Compressive strength at 28 days, chloride penetration resistance, and freeze/thaw resistance increased with incorporation of PS particles up to a 20% replacement level.
4. Polysilicon sludge particles consumed calcium hydroxide and produced additional calcium silica hydrate resulting in an improved microstructure of the concrete specimens.

## Figures and Tables

**Table 1:** Chemical composition and physical characteristics of ordinary portland cement (OPC) and polysilicon (PS).

Description	OPC	PS
SiO_2_ (%)	20.8	96.44
TiO_2_ (%)	-	0.01
Al_2_O_3_ (%)	6.3	0.03
Fe_2_O_3_ (%)	3.2	3.276
CaO (%)	62.0	0.06
Cl (%)	-	0.03
CuO (%)	-	0.07
ZnO (%)	-	0.03
MgO (%)	2.9	-
MnO (%)	-	0.007
SO_3_ (%)	2.1	0.03
Ignition loss (%)	1.5	-
Specific gravity (g/cm^3^)	3.15	1.95
Surface area (cm^2^/g)	3410	7122

**Table 2:** Physical properties of the fine and coarse aggregates.

Description	Fine Aggregate	Coarse Aggregate
G_max_ (mm)	4.75	25
G_min_ (mm)	0.075	4.75
Density (g/cm^3^)	2.62	2.66
Absorption rate (%)	1.05	0.72
Fineness modulus (FM)	2.70	6.91
Abrasion rate (AR) (%)	-	25.1
Unit volume mass (g/cm^3^)	-	1.564

**Table 3:** Properties of chemical admixtures.

-	Specific Gravity	pH (25 °C)	Cl^−^ Content (%)	Alkali Content (%)	Color/Type	Usage
SP	1.06 ± 0.05	6.5 ± 1.0	< 0.01	< 0.02	Brown/liquid	C × 0.5%
AE	1.04 ± 0.01	-	-	-	Translucent/liquid	C × 0.2%

**Table 4:** Mix proportion of concrete with PS.

Types	W/B (%)	S/A (%)	Unit Weight (kg/m^3^)						
			Water	OPC	Sand	Gravel	PS	AE	SP
Plain	40.0	48	175	337	859	919	-	0.68 (C × 0.2%)	1.69 (C × 0.5%)
PS 5%				320			17		
PS 10%				303			34		
PS 15%				286			51		
PS 20%				270			67		
PS 25%				253			84		

**Table 5:** Pozzolanic activity index (PAI) of concrete mixes.

Concrete Mix	Pozzolanic Activity Index (PAI)		
	3 days	7 days	28 days
Plain	100	100	100
PS 5%	96.2	93.0	107.6
PS 10%	94.7	91.3	106.5
PS 15%	95.7	95.3	110.4
PS 20%	98.1	100.9	116.0
PS 25%	95.7	97.9	107.5
